# Reactions of wintering passerines to male calls of the European cuckoo *Cuculus canorus*

**DOI:** 10.1038/s41598-024-64270-7

**Published:** 2024-06-20

**Authors:** Piotr Tryjanowski, Artur Golawski, Łukasz Jankowiak, Anders Pape Møller

**Affiliations:** 1 Department of Zoology, Poznań University of Life Sciences, Wojska Polskiego 71C, 60-625 Poznan, Poland; 2 Faculty of Sciences, University of Siedlce, Prusa 14, 08-110 Siedlce, Poland; 3 Department of Ecology and Anthropology, Institute of Biology, University of Szczecin, Wąska 13, 71-415, Szczecin, Poland; 4 Ecologie Systématique Evolution, Université Paris-Sud, CNRS, Université Paris-Saclay, 91405 Orsay Cedex, AgroParisTech France

**Keywords:** Calls, Nest parasitism, Passerines, Poland, Wintering, Ecology, Zoology

## Abstract

The reaction of birds to the nest parasite, the European cuckoo *Cuculus canorus*, has been the subject of extensive testing in various aspects. However, while the cuckoo is a long-distance migrant, some of its hosts are sedentary species. In this study, we aimed to investigate whether species, primarily hosts, react to the presence of the cuckoo also in the winter season. This behaviour may involve an attempt to drive the parasite away from locations that will subsequently become their breeding sites. During playback experiments conducted in the winter of 2021/2022 in Poland, we demonstrated that numerous bird species react to the male cuckoo calls in winter. These calls may be perceived as a source of danger, particularly by cuckoo hosts, who responded to this call more frequently than non-hosts and the control species (pigeon). Nonetheless, the birds’ reactions were not strong, as they did not approach the source of the call. However, our results are constrained by the limited number of cuckoo host species wintering in Poland. To better evaluate the intensity of bird responses to the male cuckoo’s call during the non-breeding season, further studies should be conducted in regions where a greater variety of species, especially those most susceptible to parasitism, overwinter.

## Introduction

The study of animal behaviour has unveiled numerous adaptive strategies that individuals employ to cope with various threats^1^. The primary threat influencing the behaviour of different taxa is the risk of predation, which has been extensively documented e.g.^2–5^, as well as food availability^6–8^. Another critical threat impacting animal behaviour is parasitism, which encompasses brood parasitism^9–11^.

Brood parasitism represents a unique reproductive strategy where avian brood parasites lay their eggs in the nests of other birds, thereby transferring the reproductive costs to the hosts^11^. These costs compel hosts to develop various anti-parasitic strategies, such as rejecting parasite eggs upon recognition^12–14^ or abandoning the brood^15^. In response, brood parasites have evolved countermeasures, such as mimicking the eggs or nestlings of their hosts^16,17^. Hosts’ behavioural responses to parasitism threats also include aggressive mobbing against parasites, which may involve physical contact and pose risks to both the parasite and host^18,19^.

It is crucial for the host’s fitness to be able to recognize its parasite^20^. The ability of hosts to distinguish between the European cuckoo (*Cuculus canorus*), a well-known parasite of small passerines in Europe and Asia, is often assessed by observing their behavioural reactions to dummies of cuckoos and other species, including the predatory sparrowhawk (*Accipiter nisusus*)^24^. Parasites, in response to host attacks, have evolved coloration that mimics predators threatening the hosts. A significant number of species fail to differentiate the cuckoo from the sparrowhawk, reacting with comparable aggression towards both, yet showing greater hostility than towards control species, primarily pigeons^21,22^. However, some studies suggest that certain species can distinguish between a cuckoo and a sparrowhawk but see Ref^23,24^, and exhibit aggressive behavior towards the cuckoo, sometimes resulting in its death^25^. Even non-host species, such as tits, substantially reduce their activity at feeders in the presence of both cuckoo and sparrowhawk dummies. Additionally, there is a likelihood that birds confuse the calls of the female cuckoo and the sparrowhawk^26,27^. These findings strongly indicate that at least some species may mistake common cuckoos for sparrowhawks, with the visual and vocal similarities of the common cuckoo to the sparrowhawk being considered an example of predator mimicry^21,28^. However, this raises the question of what exactly is being tested with a dummy or the voice of a female cuckoo: the response to the sparrowhawk or to the cuckoo? It appears that despite numerous studies, our understanding of the parasite-host system remains limited^29^.

Host or cuckoo responses were primarily observed during the egg-laying period and early incubation, when the threat to the hosts’ broods was most realistic^14,30^. It is plausible that some hosts may exhibit similar levels of aggression towards the cuckoo from egg-laying through the nestling period^31,32^, suggesting that birds’ aggressive behaviour towards the cuckoo may extend beyond periods of direct threats to breeding. However, data on bird reactions to the cuckoo during the non-breeding period are scarce and primarily based on the presentation of a dummy or the voice of a female cuckoo^21,33^.

In this study, we expand the traditional coevolutionary framework that has characterized previous research. We examine the extent to which species respond to the voice of the male cuckoo during winter. The male cuckoo’s call is more frequent and thus more readily available to birds than the female’s call. The greater availability of cues based on male calls makes them potentially valuable for bird species, allowing them to avoid areas where the parasite is present^34,35^. It has also been established that birds respond to the call of a male cuckoo^33,36^. We hypothesized that the host’s reaction to male cuckoo calls should be stronger than their reaction to pigeon calls (control species) and also stronger than reactions of non-hosts. The cuckoo is a breeding species in Poland, obligatorily migrating away for winter, whereas the woodpigeon occasionally winters in Poland but was not recorded in the study area during this time^37,38^. An additional objective was to determine whether winter conditions, time of day, and temperature influence the reaction. We also investigated regional differences in reactions by conducting experiments in two areas with varying winter severity and, consequently, distinct compositions of wintering bird species assemblages.

## Methods

### Study areas

Fieldwork was conducted in two study locations: one in eastern and one in western Poland. The eastern study area, located in Podlasie (P) near Siedlce (center of the area: 52° 10′ N, 22° 16′ E), was predominantly characterized by agricultural landscapes, including arable fields and meadows extensively utilized by farmers. The woodlands in this region were either exclusively coniferous, deciduous, or mixed. Hedgerows consisted of rows or clusters of trees. Additionally, small watercourses and fishponds were present. The study in western Poland was conducted in an agriculturally intensive landscape in Wielkopolska (W) near Poznań (center of the area: 52° 24′ N, 16° 54′ E). The agricultural intensity in Wielkopolska was notably greater than in the Podlasie region, with larger fields and species-diverse woodlands and hedgerows.

The climates of these two areas differ, with winters in western Poland being milder than those in the eastern part^39^. From December 2021 to February 2022, the mean daily air temperature in Podlasie was 0.4 °C, with a minimal daily air temperature of − 2.4 °C, and total precipitation of 109.5 mm. Corresponding data for Wielkopolska were: 1.8 °C, − 1.5 °C, and 154.9 mm (source: http://www.tutiempo.net for meteorological stations in Siedlce and Poznań, respectively). Likely due to climatic factors, winter bird assemblages in eastern Poland were less diverse and fewer in number^37,38^.

### Playback experiments

For our playback experiments, we used high-quality samples of local territorial calls from the common cuckoo and the common woodpigeon for control purposes. To avoid pseudo-replication, we randomly selected three different calls from each species based on their quality. We chose male calls for their longer duration and the greater responsiveness of birds to these calls^33^. The playback sounds were filtered and standardized to achieve a natural amplitude within the loudspeakers using Avisoft SASLab Pro 5.2.x. Each playback sequence began with 120 s of silence to allow for setup and observer departure, followed by the specific call pattern. (1) Treatment (cuckoo): A basic unit of 20 cuckoo calls was played over 25 s, repeated five times with 35-s intervals. (2) Control (woodpigeon, *Columba palumbus*): The playback pattern mirrored the cuckoo’s, but the basic unit consisted of 16 woodpigeon calls over 25 s. Each experimental playback lasted 5 min. The calls were broadcast through waterproofed Blaupunkt BT12 Outdoor loudspeakers, with the amplitude standardized across all playbacks to maintain natural levels (80–95 dB SPL at 1 m)^36,40^.

### Experimental design

Experiments were conducted during winter, from December 2021 to February 2022. A total of 230 experimental trials were carried out under favourable weather conditions (no snow, rain, or strong wind). In Podlasie, 96 trials were conducted (49 cuckoo and 47 woodpigeon calls), and 134 trials in Wielkopolska (67 for each call type), each at a different location. The minimum distance between two adjacent experimental sites was 1 km, and the observer alternated the playback of the two species’ calls at successive sites. A minimum interval of 20 min was maintained between two sound playbacks. Observers, with over 30 years of birding experience) approximately 30 m from the loudspeaker and counted all birds seen or heard within a 100-m radius, resulting in a maximum bird identification range of 130 m.

### Data analyses

Experiments were categorized based on background counts into two types: background host bird presence and their reaction to cuckoo and pigeon calls, and non-host bird background presence and their reaction to cuckoo and pigeon calls. We included experiment records only when at least one host or non-host species was present in the background, except in cases where a species was not recorded in the background counts but a reaction was observed in the experiment. The hosts of the cuckoo were classified according to the most frequently parasitized species recorded in Poland^41–43^. As the dependent variable, we used the proportion of reaction presence to the number of species in the background (expressed as the summed species presence) to the presented calls. We defined a reaction presence to the calls as observed when a bird exhibited a nervous reaction or approached the direction of the loudspeaker. Owing to the predominantly weak nature of the birds’ reactions, criteria describing the strength of these reactions were omitted, and we retained the term “response recorded”. Most reactions were performed by a single individual of a species. In only four instances, more than one individual exhibited a reaction. Therefore, we analyzed these cases as the total occurrence of reactions within a given species (using a binomial model) rather than counting the number of reactions (which would have required a Poisson model). This approach was also applied to the number of individuals of each species present in the background, analyzing the summed presence of each species rather than evaluating the summed species abundance. Further details can be found in the Supplementary Materials, Table S1.

Since the calls of cuckoos and pigeons were presented to distinct groups of background species, the independent variable ‘trial’ was categorized into four levels: CU_Host (cuckoo call exposed to host species), CP_Host (pigeon call exposed to host species), CU_Non-host (cuckoo call exposed to non-host species), and CP_Non-host (pigeon call exposed to non-host species). Other independent variables used in the model were temperature, day of winter, and hour.

The significance of the terms of the GLM model was tested using a likelihood ratio test (LRT), which compared the Akaike Information Criterion (AIC) of the full model to a reduced model where the tested variable had been dropped. All statistical analyses were performed using the R software^44^. Post-hoc analyses using Sidak tests were conducted in the R package *emmeans*^45^.

In the subsequent analysis, instead of grouping species into non-host and host categories, we used phylogenetic analysis. Initially, 100 trees were downloaded based on Ericson et al.’s^46^ backbone phylogeny. These trees were then summarized according to Rubolini’s guidelines^47^ to obtain an optimal consensus tree (using the 50% majority-rule) with the SumTrees application incorporated in the DendroPy library in Python^48^. To account for phylogenetic relationships, we used a phylogenetic generalized linear mixed model with a binomial distribution, performed using the package “phyr”^49^ This model incorporated the phylogenetic covariance matrix, adding the optimal consensus tree to the standard mixed model, implying that closely related birds might have similar intercepts. The phylogenetic trees used were downloaded from https://birdtree.org/^50^. The significance of the phylogenetic effect was tested using a likelihood ratio test (LRT).

### Ethical approval

This study, being observational in nature, did not necessitate formal consent under Polish national law for this type of research. Additionally, our study did not require approval from the Local Ethical Commission as playback experiments are not under its jurisdiction in Poland, according to The Act on Experiments on Animals (Disposition no. 289 from 2005). To minimize potential disturbances, the playback duration was kept as brief as necessary for data collection. Furthermore, the experiments were conducted outside the breeding season of the birds, and to the best of our knowledge, there have been no adverse effects on the subjects’ breeding or welfare.

## Results

During the winter experiments, we recorded a total of 836 individuals, representing 49 species (see Supplementary Material). The most commonly observed species in the background were the great tit (*Parus major*), the blue tit (*Cyanistes caeruleus*), and the jay (*Garrulus glandarius*), as detailed in the Supplementary Material (Table S1).

In total, we observed 20 reactions from five host species to cuckoo calls and 16 reactions from ten non-host species (Supplementary Material, Table S2). For pigeon calls, two reactions were noted for two host species and two reactions for two non-host species.

**Figure 1:**
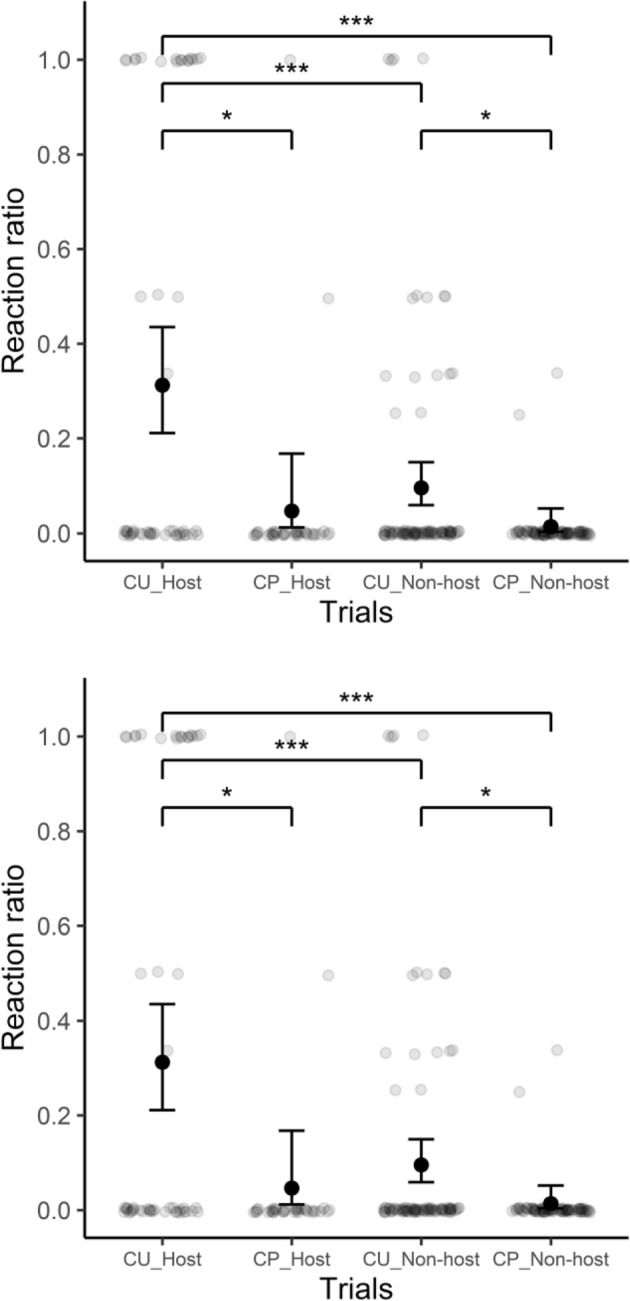
Influence of cuckoo (CU) and pigeon (CP) calls on the reaction ratio in host and non-host species. The reaction ratio is calculated as the proportion of individual responses per species within each trial group, where a value of 1 indicates that all observed individuals reacted, while a value of 0 indicates no reactions were observed. Whiskers represent 95% confidence intervals. Significant post hoc differences between groups are indicated with asterisks (***p < 0.001, *p < 0.05).

The ‘trials’ variable was significant (LRT = 41.550, df = 3, *p* < 0.001). Post-hoc comparisons indicated a difference in reactions between the trials conducted on host species: the probability of host species reacting was higher to cuckoo calls (CU_Host; 0.31 ± 0.058 s.e.) than to pigeon calls (CP_Host: 0.05 ± 0.032 s.e.; CU_Host vs. CP_Host, z-ratio = 2.88, *p* = 0.020). A similar pattern was observed in experimental trials of cuckoo and pigeon calls when presented to non-host species (CU_Non-host: 0.10 ± 0.022 s.e.; CP_Non-host: 0.01 ± 0.009 s.e.; CU_Non-host vs. CP_Non-host, z-ratio = 2.697, *p* = 0.035). Post-hoc comparisons revealed a higher probability of reaction in host species to cuckoo calls than in non-host species to cuckoo calls (CU_Host vs. CU_Non-host, z-ratio = 3.885, *p* < 0.001). Comparisons between the reaction of hosts to cuckoo calls and that of non-hosts to pigeon calls also indicated a higher reaction in hosts to cuckoo calls (CU_Host vs. CP_Non-host; z-ratio = 4.610, *p* < 0.001). Other comparisons, such as CP_Host vs CP_Non-host and CP_Host versus CU_Non-host, showed no statistical differences (*p* > 0.05 for both comparisons; Fig. 1).

The temperature variable was not significant (LRT = 0.233, df = 1, *p* = 0.629). Similarly, the day of winter was not a significant factor (LRT = 0.139, df = 1, *p* = 0.709). The hour of the experiment was also found to be insignificant (LRT = 0.439, df = 1, *p* = 0.507), and no significant differences were observed between the study regions (LRT = 0.337, df = 1, *p* = 0.562).

We found the phylogenetic effect to be significant (LRT = 19.623, df = 1, *p* < 0.001). For detailed model parameters, please refer to the Supplementary materials.

## Discussion

We tested the effect of the male cuckoo call on the responses of passerine birds during winter. Our experimental findings revealed that bird responses to male cuckoo calls were significantly stronger in host species compared to non-host species, as well as to the call of the pigeon, which served as a control species. Additionally, we observed that non-host species exhibited a slightly stronger response to the cuckoo call compared to their response to the control pigeon call. Importantly, we did not find that the birds’ reactions to the cuckoo call were influenced by the progression of winter, the time of the experiment, or the location of the study area. Thus, our most significant discovery is the markedly stronger reaction of host species to male cuckoo calls.

Given that this is the first study of its kind conducted in winter, we lack relevant reference data for comparison. However, our results can be related to similar experiments involving the playback of cuckoo calls to four bird species (two host species and two potential host species) conducted in winter in China. That study found that birds responded more strongly to the voice of a female cuckoo than to that of a male cuckoo, with no differences observed between the four species tested^33^. The authors of that study, however, questioned the applicability of their results to other bird species. Another study conducted in the UK during winter used cuckoo dummies^21^, but the use of physical models makes direct comparisons with our findings challenging. It is known that birds, including non-host species, may attack cuckoos outside the breeding season, a behaviour attributed to the cuckoo’s resemblance to sparrowhawks^51^. Additionally, in studies on *Chalcites* cuckoo species, hosts showed concern for the voice of parasites during the non-breeding season, which was interpreted as an attempt to drive the parasite away from their future breeding grounds^17^.

Our results suggest that the male cuckoo’s call is perceived by some species as a threat, potentially extending from the breeding season, as previously demonstrated^52^. This hypothesis is supported by the fact that cuckoo hosts reacted much more strongly than non-host species. Parasites can be treated by their prey (host species) as a kind of predator. Several studies have shown that prey responds to predators by adopting behaviours indicative of danger from a specific predator^53,54^. During the non-breeding period, when there is no threat to the brood, these species may indicate the presence of the parasite through its call. Even a relatively weak response could alert other species to potential danger, in line with the “Alerting Others Hypothesis” of enemy recognition in birds^55^. Birds that have encountered a specific threat, e.g., a cuckoo, may retain this memory throughout their lives^56^. However, we were surprised to find that the reaction of non-hosts to the male cuckoo voice was clearly discernible. Perhaps these non-host species also perceive the cuckoo as a species that causes anxiety in other birds, thereby associating its call with danger to some extent. For these species, visual confirmation may be necessary to further ascertain the threat^57^.

## Conclusions

Our findings indicate that the call of the male cuckoo is perceived by birds as a potential source of danger, even in winter. This is particularly evident among cuckoo hosts, who exhibited more frequent responses to this call compared to non-hosts and the control species (pigeon). However, the birds’ reactions were generally subdued; they did not actively approach the source of the sound. This restrained behaviour was likely due to the absence of broods during the winter period. Nevertheless, our conclusions are constrained by the limited number of cuckoo host species wintering in Poland. For a more comprehensive assessment of the birds’ responses to the call of the male cuckoo during the non-breeding season, further studies should be conducted in regions where a greater variety of species, particularly those highly susceptible to parasitism, are present during winter.

### Supplementary Information

Supplementary Information 1.

Supplementary Information 2.

Supplementary Information 3.
