# Supplementary material for: Reactions of wintering passerines to male calls of the European cuckoo Cuculus canorus
Source: Sci Rep. 2024 Jun 20;14:14204. doi: 10.1038/s41598-024-64270-7 (PMC11189894; doi:10.1038/s41598-024-64270-7)
Supplement: Supplementary file 1 — Supplementary Information 1. [file 41598_2024_64270_MOESM1_ESM.docx]

**Supplementary Materials**

Table S1. Summed species abundance and species presence of host and non-host species of background counts.

|  | | | | Abundance | | | Presence | | |
| --- | --- | --- | --- | --- | --- | --- | --- | --- | --- |
|  |  |  |  | **Location** | | **Sum** | **Location** | | **Sum** |
| Nr | **Type** | **Common name** | **Scientific name** | **P** | **W** |  | **P** | **W** |  |
| 1 | Host | Goldfinch | *Carduelis carduelis* | 7 | 4 | 11 | 3 | 1 | 4 |
| 2 | Host | Greenfinch | *Chloris chloris* | 2 | 0 | 2 | 1 | 0 | 1 |
| 3 | Host | Hawfinch | *Coccothraustes coccothraustes* | 1 | 3 | 4 | 1 | 4 | 5 |
| 4 | Host | Yellowhammer | *Emberiza citrinella* | 20 | 77 | 97 | 8 | 10 | 18 |
| 5 | Host | Reed Bunting | *Emberiza schoeniclus* | 0 | 1 | 1 | 0 | 1 | 1 |
| 6 | Host | European Robin | *Erithacus rubecula* | 0 | 1 | 1 | 0 | 1 | 1 |
| 7 | Host | Chaffinch | *Fringilla coelebs* | 1 | 25 | 26 | 1 | 13 | 14 |
| 8 | Host | Great Grey Shrike | *Lanius excubitor* | 1 | 1 | 2 | 1 | 1 | 2 |
| 9 | Host | Linnet | *Linaria cannabina* | 0 | 17 | 17 | 0 | 2 | 2 |
| 10 | Host | Eurasian Bullfinch | *Pyrrhula pyrrhula* | 9 | 21 | 30 | 7 | 5 | 12 |
| 11 | Host | Goldcrest | *Regulus regulus* | 0 | 4 | 4 | 0 | 3 | 3 |
| 12 | Host | Stonechat | *Saxicola rubicola* | 0 | 1 | 1 | 0 | 1 | 1 |
| 13 | Host | Blackcap | *Sylvia atricapilla* | 0 | 1 | 1 | 0 | 1 | 1 |
| 14 | Host | Wren | *Troglodytes troglodytes* | 2 | 2 | 4 | 2 | 2 | 4 |
| 15 | Host | Blackbird | *Turdus merula* | 2 | 19 | 21 | 2 | 19 | 21 |
| 1 | Non-host | Sparrowhawk | *Accipiter nisus* | 1 | 0 | 1 | 1 | 0 | 1 |
| 2 | Non-host | Long-tailed Tit | *Aegithalos caudatus* | 31 | 6 | 37 | 4 | 2 | 6 |
| 3 | Non-host | Mallard | *Anas platyrhynchos* | 0 | 4 | 4 | 0 | 1 | 1 |
| 4 | Non-host | Bittern | *Botaurus stellaris* | 0 | 1 | 1 | 0 | 1 | 1 |
| 5 | Non-host | Buzzard | *Buteo buteo* | 0 | 6 | 6 | 0 | 5 | 5 |
| 6 | Non-host | Short-toed Tree-Creeper | *Certhia brachydactyla* | 0 | 2 | 2 | 0 | 2 | 2 |
| 7 | Non-host | Tree-Creeper | *Certhia familiaris* | 0 | 3 | 3 | 0 | 3 | 3 |
| 8 | Non-host | Raven | *Corvus corax* | 28 | 16 | 44 | 13 | 12 | 25 |
| 9 | Non-host | Hooded Crow | *Corvus cornix* | 0 | 4 | 4 | 0 | 2 | 2 |
| 10 | Non-host | Blue Tit | *Cyanistes caeruleus* | 24 | 30 | 54 | 13 | 29 | 42 |
| 11 | Non-host | Great Spotted Woodpecker | *Dendrocopos major* | 5 | 22 | 27 | 5 | 21 | 26 |
| 12 | Non-host | Middle Spotted Woodpecker | *Dendrocoptes medius* | 1 | 2 | 3 | 1 | 2 | 3 |
| 13 | Non-host | Lesser Spotted Woodpecker | *Dryobates minor* | 0 | 4 | 4 | 0 | 4 | 4 |
| 14 | Non-host | Black Woodpecker | *Dryocopus martius* | 3 | 3 | 6 | 3 | 3 | 6 |
| 15 | Non-host | Corn Bunting | *Emberiza calandra* | 0 | 47 | 47 | 0 | 3 | 3 |
| 16 | Non-host | Merlin | *Falco columbarius* | 0 | 1 | 1 | 0 | 1 | 1 |
| 17 | Non-host | Crested Lark | *Galerida cristata* | 0 | 2 | 2 | 0 | 1 | 1 |
| 18 | Non-host | Jay | *Garrulus glandarius* | 13 | 22 | 35 | 13 | 21 | 34 |
| 19 | Non-host | Crane | *Grus grus* | 0 | 3 | 3 | 0 | 2 | 2 |
| 20 | Non-host | White-tailed Eagle | *Haliaeetus albicilla* | 0 | 1 | 1 | 0 | 1 | 1 |
| 21 | Non-host | Crested Tit | *Lophophanes cristatus* | 0 | 2 | 2 | 0 | 2 | 2 |
| 22 | Non-host | Bearded Reedling | *Panurus biarmicus* | 0 | 1 | 1 | 0 | 1 | 1 |
| 23 | Non-host | Great Tit | *Parus major* | 18 | 52 | 70 | 11 | 45 | 56 |
| 24 | Non-host | Tree Sparrow | *Passer montanus* | 2 | 6 | 8 | 1 | 5 | 6 |
| 25 | Non-host | Coal Tit | *Periparus ater* | 0 | 3 | 3 | 0 | 3 | 3 |
| 26 | Non-host | Pheasant | *Phasianus colchicus* | 1 | 0 | 1 | 1 | 0 | 1 |
| 27 | Non-host | Magpie | *Pica pica* | 6 | 14 | 20 | 5 | 14 | 19 |
| 28 | Non-host | Green Woodpecker | *Picus viridis* | 0 | 3 | 3 | 0 | 3 | 3 |
| 29 | Non-host | Marsh Tit | *Poecile palustris* | 5 | 3 | 8 | 5 | 3 | 8 |
| 30 | Non-host | Nuthatch | *Sitta europaea* | 1 | 14 | 15 | 1 | 13 | 14 |
| 31 | Non-host | Siskin | *Spinus spinus* | 13 | 90 | 103 | 4 | 4 | 8 |
| 32 | Non-host | Collared Dove | *Streptopelia decaocto* | 0 | 3 | 3 | 0 | 4 | 4 |
| 33 | Non-host | Fieldfare | *Turdus pilaris* | 12 | 33 | 45 | 3 | 5 | 8 |
| 34 | Non-host | Mistle Thrush | *Turdus viscivorus* | 0 | 5 | 5 | 0 | 5 | 5 |

Location – P: Podlasie region (eastern Poland), W: Wielkopolska (western Poland)

Table S2. Summed reaction amount and reaction presence of host and non-host species during the experiment.

| Order number | Type | Common name | Scientific name | Summed reaction presence | Summed reaction amount | Summed reaction presence to cuckoo calls | Summed reaction presence to pigeon calls |
| --- | --- | --- | --- | --- | --- | --- | --- |
| 1 | Host | Wren | *Troglodytes troglodytes* | 9 | 9 | 8 | 1 |
| 2 | Host | European Robin | *Erithacus rubecula* | 7 | 7 | 6 | 1 |
| 3 | Host | Blackbird | *Turdus merula* | 4 | 4 | 4 | 0 |
| 4 | Host | Yellowhammer | *Emberiza citrinella* | 1 | 1 | 1 | 0 |
| 5 | Host | Blackcap | *Sylvia atricapilla* | 1 | 1 | 1 | 0 |
| 1 | Non-host | Great Tit | *Parus major* | 2 | 8 | 2 | 0 |
| 2 | Non-host | Blue Tit | *Cyanistes caeruleus* | 5 | 5 | 5 | 0 |
| 3 | Non-host | Long-tailed Tit | *Aegithalos caudatus* | 2 | 3 | 2 | 0 |
| 4 | Non-host | Tree Sparrow | *Passer montanus* | 2 | 3 | 2 | 0 |
| 5 | Non-host | Crested Lark | *Galerida cristata* | 1 | 2 | 1 | 0 |
| 6 | Non-host | Jay | *Garrulus glandarius* | 2 | 2 | 1 | 1 |
| 7 | Non-host | Bearded Reedling | *Panurus biarmicus* | 1 | 1 | 1 | 0 |
| 8 | Non-host | Goshawk | *Accipiter gentilis* | 1 | 1 | 1 | 0 |
| 9 | Non-host | Sparrowhawk | *Accipiter nisus* | 1 | 1 | 1 | 0 |
| 10 | Non-host | Nuthatch | *Sitta europaea* | 1 | 1 | 0 | 1 |

Table S3. Estimates of models explaining reaction probability.

| Fixed | Value | Std.Error | Zscore | Pvalue |
| --- | --- | --- | --- | --- |
| (Intercept) | -5.026 | 2.532 | -1.985 | 0.047 |
| Trial CU | 2.368 | 0.594 | 3.989 | <0.001 |
| Temp | -0.058 | 0.060 | -0.955 | 0.340 |
| dow | 0.006 | 0.017 | 0.375 | 0.707 |
| hour | 0.015 | 0.179 | 0.083 | 0.934 |
| random | Variance | Std.Dev |  |  |
| 1\|species | <0.001 | 0.017 |  |  |
| 1\|species_phylo | 2.144 | 1.464 |  |  |
| 1\|sites | 0.000 | 0.008 |  |  |
| 1\|species_phyl@site | <0.001 | 0.004 |  |  |

Note: species and sites – random effect indication the variation between species and site; species_phylo - random effect of species with phylogenetic relationships among them; species_phylo@site – nested random effect: close related species tend to be in the same site; dow – day of week; Trial – CU (cuckoo call exposed) vs CP (pigeon call exposed)
